# CircINSR Regulates Fetal Bovine Muscle and Fat Development

**DOI:** 10.3389/fcell.2020.615638

**Published:** 2021-01-06

**Authors:** Xuemei Shen, Jia Tang, Wenxiu Ru, Xiaoyan Zhang, Yongzhen Huang, Chuzhao Lei, Hui Cao, Xianyong Lan, Hong Chen

**Affiliations:** ^1^Key Laboratory of Animal Genetics, Breeding and Reproduction of Shaanxi Province, College of Animal Science and Technology, Northwest A&F University, Yangling, China; ^2^Shaanxi Kingbull Livestock Co., Ltd., Yangling, China

**Keywords:** bovine, muscle development, circular RNAs, cell proliferation, preadipocyte

## Abstract

The level of muscle development in livestock directly affects the production efficiency of livestock, and the contents of intramuscular fat (IMF) is an important factor that affects meat quality. However, the molecular mechanisms through which circular RNA (circRNA) affects muscle and IMF development remains largely unknown. In this study, we isolated myoblasts and intramuscular preadipocytes from fetal bovine skeletal muscle. Oil Red O and BODIPY staining were used to identify lipid droplets in preadipocytes, and anti-myosin heavy chain (MyHC) immunofluorescence was used to identify myotubes differentiated from myoblasts. Bioinformatics, a dual-fluorescence reporter system, RNA pull-down, and RNA-binding protein immunoprecipitation were used to determine the interactions between circINSR and the micro RNA (miR)-15/16 family. Molecular and biochemical assays were used to confirm the roles played by circINSR in myoblasts and intramuscular preadipocytes. We found that isolated myoblasts and preadipocytes were able to differentiate normally. CircINSR was found to serve as a sponge for the miR-15/16 family, which targets *CCND1* and *Bcl-2*. CircINSR overexpression significantly promoted myoblast and preadipocyte proliferation and inhibited cell apoptosis. In addition, circINSR inhibited preadipocyte adipogenesis by alleviating the inhibition of miR-15/16 against the target genes *FOXO1* and *EPT1*. Taken together, our study demonstrated that circINSR serves as a regulator of embryonic muscle and IMF development.

## Introduction

In livestock production, the development of muscle and intramuscular fat (IMF) are important factors that determine meat quality. Bovine muscle development begins during the early embryonic stage, including the proliferation of myoblast progenitor cells and the proliferation and fusion of mononuclear myoblasts to form multinucleated myotubes. After birth, the number of muscle fibers does not change, but the fibers become thicker (Rehfeldt et al., 2000; Picard et al., 2010). Therefore, the level of muscle development that occurs during pregnancy directly affects meat production. Fat begins to develop during the second trimester (Sanchez-Gurmaches and Guertin, 2014). The IMF content determines the tenderness and juiciness of beef, and the optimization of this factor is a research hotspot. Increasing the number of preadipocytes in fetal muscle contributes to improved fat deposition and marbling after birth (Rajesh et al., 2010). However, the mechanisms that control muscle and IMF development remain unclear. In addition, pre-mature adipogenesis and the maturation of intramuscular preadipocytes in the fetus can result in muscle tissue dysfunction (Taga et al., 2011). Therefore, exploring the molecular mechanisms that regulate muscle and IMF development is of great significance to the livestock industry.

The development of biotechnology has greatly promoted the ability to screen and study key genes involved in muscle and IMF development. Several critical genes have been demonstrated to mediate muscle development and adipogenesis, including peroxisome proliferative activated receptor gamma (*PPAR*γ) and CCAAT/enhancer-binding protein alpha (*C/EBP*α), which have been characterized as adipogenesis regulators (Lin and Lane, 1994; Tontonoz et al., 1994). The myogenic regulatory factors (*MRFs*), myocyte enhancer factor (*MEF*2), *PAX3/PAX7*, and myostatin (*MSTN*) have been reported to be effective for the induction of myoblasts proliferation and differentiation (Sassoon et al., 1989; McPherron and Lee, 1997; Soumillion et al., 1997; Relaix et al., 2005; Potthoff and Olson, 2007).

In addition to coding genes, a large number of non-coding RNAs have also been shown to regulate muscle and fat development. For example, a large number of micro RNAs (miRNAs) and circular RNAs (circRNAs) have been reported to participate in the physiological regulation of muscle and fat. CircRNAs have a covalent closed-loop structure, with neither 5′-3′ polarity nor a polyadenylated tail (Hansen et al., 2013a). They can participate in physiological regulation by sponging miRNAs or binding regulatory proteins (Meng et al., 2017). CircFUT10 and circFGFR4 have been reported to regulate the muscle development-related genes through the sponging of miR-133a and miR-107 (Li et al., 2018a,b). CircINSR has been shown to adsorb miR-34a to promote the proliferation of bovine myoblasts (Shen et al., 2020). CircTshz2-1 and circArhgap5-2 have been shown to be indispensable regulators of fat formation (Arcinas et al., 2019). Despite these findings, the roles played by circRNAs in muscle and IMF development continues to require additional research. Based on our previous studies, we have focused on a circRNA derived from the insulin receptor gene *(INSR)* in this study. CircINSR is named after the *INSR* gene and is formed by the cyclization of the second exon of *INSR* (Shen et al., 2020). A previous study indicated that circINSR could affect the proliferation and apoptosis of bovine embryonic myoblasts by adsorbing miR-34a; however, whether circINSR interacts with other miRNAs and whether circINSR plays a role in preadipocyte development remains unclear.

In this study, we isolated fetal bovine myoblasts and intramuscular preadipocytes. The targeting relationship between circINSR and the miR-15/16 family and the effects of circINSR on the proliferation and apoptosis of myoblasts and preadipocytes were analyzed *in vitro*. Importantly, we demonstrated that circINSR could target miR-15/16 to inhibit the pre-mature differentiation of intramuscular preadipocytes, ensuring normal muscle function.

## Materials and Methods

### Progenitor Cell Isolation and Cell Lines

Bovine fetuses, from 120 to 180 days, were collected from the slaughterhouse and transported immediately to the laboratory. All animal experiments and study protocols were approved by the Animal Care Commission of the College of Veterinary Medicine, Northwest A&F University. Using enzyme digestion, combined with the differential adhesion method, primary myoblasts and intramuscular preadipocytes were isolated from the *longissimus dorsi*, as previously described (Rando and Blau, 1994; Conboy and Rando, 2002; Miyake et al., 2012). The *longissimus dorsi* was isolated from the fetus, washed with phosphate-buffered saline (PBS), and minced into small fragments. It was then digested in Dulbecco's modified Eagle's medium (DMEM, HyClone, USA) containing type II collagenase (w/v, 2%; C5138, Sigma, USA) at 37°C, with continuous shaking for 2 h. The cell plasma was filtered through a 200-μm filter, collected by centrifugation, and resuspended in DMEM. The cells were grown in high-glucose DMEM supplemented with 20% fetal bovine serum (FBS, Gibco, USA) and 1% penicillin-streptomycin solution (Gibco, USA), and incubated at 37°C with 5% CO_2_. Within 2 h of inoculation, the adherent cells were collected for adipogenesis induction. The supernatant containing non-adherent cells was cultured for an additional 24 h, after which the supernatant was collected and cultured in a new dish. After repeating this process for 2 days, the adherent cells were collected for myogenesis induction. HEK-293T cells were purchased from the American Type Culture Collection (ATCC) and were verified to be negative for mycoplasma contamination.

### Differentiation of Myoblasts and Preadipocytes

Myoblasts were cultured in high-glucose DMEM, supplemented with 20% FBS. Two days after cells reached confluence, DMEM containing 2% horse serum (HyClone, USA) and 1% penicillin-streptomycin solution was used for myogenesis induction. Myotube identification was performed on the 4th day of induction. Intramuscular preadipocytes were cultured in DMEM/F12 (HyClone, USA) supplemented with 10% FBS and 1% penicillin-streptomycin solution. Adipocyte differentiation was induced by M1 medium [DMEM/F12 containing 10% FBS, 1% penicillin-streptomycin solution, 0.5 mM 3-isobutyl-1-methylxanthine (Sigma, USA), 1 μM dexamethasone (Sigma, USA), and 1.5 μg/mL insulin (Sigma, USA)]. Two days later, the M1 medium was replaced with M2 medium (DMEM/F12 containing 10% FBS, 1% penicillin-streptomycin solution, and 1.5 μg/mL insulin). Then, differentiation was induced for 8 days, during which time the medium was changed once every 2 days. Oil Red O staining was performed on the 8th day of adipogenic differentiation.

### RNA Extraction and Real-Time qPCR

Total RNA was isolated with Trizol reagent (Invitrogen, Carlsbad, CA, USA) and cDNA was synthesized with PrimeScript™ RT reagent kit with gDNA Eraser (Takara, Tokyo, Japan). Real-time qualitative polymerase chain reaction (qPCR) for RNA analyses were performed using the SYBR Green PCR Master Mix (Takara, Tokyo, Japan). MiRNA-specific stem-loop primers were used for reverse transcription. The level of glyceraldehyde 3-phosphate dehydrogenase (GAPDH) was used to normalize the expression levels of circRNAs and mRNAs, and the level of small nuclear U6 was used to normalize the expression levels of miRNAs. The primers used in the analysis are shown in Supplementary Table 1.

### Vector Construction and Cell Transfection

The second exon sequence of the *INSR* gene was inserted into the pCD2.1 vector (Geneseed Biotech, Guangzhou, China) and psi-CHECK2 vector (Promega, Fitchburg, WI, USA). Small interfering RNA (siRNA) oligonucleotides were designed to combine with the back-splice region of circINSR (RiboBio, Guangzhou, China). These siRNAs inhibited the expression of circINSR after transfection into the cell and was named si-circINSR. The mimics of bta-miR-15a, bta-miR-15b, bta-miR-16a, and bta-miR-16b were purchased from RiboBio (Guangzhou, China). The 3′-untranslated regions (UTRs) of the cyclin D1 (*CCND1*) and B-cell lymphoma 2 (*Bcl-2*) genes containing the miR-15/16 binding sites were amplified using the PCR enzyme mix (Platinum II Taq Hot-Start DNA Polymerase, Invitrogen). The wild-type and mutant 3'-UTR gene sequences were cloned into the psi-CHECK2 vector. The Renilla: Firefly ratio was measured and compared against that for the non-treated control. The mimics (50 nM) or vectors (2 μg/mL) were transfected into cells using a transfection reagent (R0531, Thermo Fisher Scientific, USA). For the overexpression of the miR-15/16 family, the miR-15a, miR-15b, miR-16a, and miR-16b mimics were mixed in equal amounts for transfection.

### Oil Red O and BODIPY Staining

After 8 days of differentiation, the intramuscular preadipocytes were stained with Oil Red O (#O0625, Sigma, USA) and 4,4-difluoro-1,3,5,7,8-pentamethyl-4-bora-3a,4a-diaza-s-indacene (BODIPY 493/503; D3922, Thermo Fisher Scientific). Oil Red O staining was performed according to the manufacturer's instructions. To quantify the staining of fat droplets, 100% isopropanol was used to dissolve the lipid droplets, and the absorbance was measured at 510 nm. For BODIPY staining, the cells were washed twice with PBS to remove residual 4% paraformaldehyde. Hank's Balanced Salt Solution containing 10 μM BODIPY 493/503 was added to the cells and then incubated at 37°C for 30 min in the dark. The samples were washed three times with PBS and photographed immediately.

### Immunofluorescence Analysis

After 4 days of myogenic differentiation, myoblasts were fixed with 4% paraformaldehyde. After washing with PBS, myosin heavy chain (MyHC) antibody (1:250, Heavy chain cardiac Myosin antibody, GTX20015, GeneTex, USA) was incubated with the cells overnight at 4°C. The goat anti-mouse IgG (H&L) -Alexa Fluor 594 (1:500; RS3608; Immunoway Biotechnology, USA) was used as a secondary antibody. After the secondary antibody was diluted with PBS containing 1% bovine serum albumin, it was added to the cells and incubated for 2 h at room temperature. The nucleus was stained with 4′,6-diamidino-2-phenylindole (DAPI, 1:500, #62248, Thermo, USA). Finally, we washed the cells three times with PBS and observed them under a fluorescence microscope (DM5000B; Leica, Germany). The myotube coverage area was analyzed by Image-Pro Plus software.

### Dual-Luciferase Reporter Assay

HEK-293T cells were co-transfected with miR-15/16 mimics and plasmids. After 24 h of transfection, luciferase activity was detected using the Dual-Luciferase Reporter Assay Kit (#E2920, Promega, Fitchburg, WI, USA). The optical density of the resulting solution was assessed using an automatic microplate reader (Molecular Devices, Sunnyvale, USA).

### RNA Fish

RNA fluorescence *in situ* hybridization (RNA-FISH) probes that specifically recognize the back-splicing junction region of circINSR were designed (RiboBio, Guangzhou, China). Primary myoblasts and preadipocytes were fixed with *in situ* hybridization fixative. After pre-hybridization, the cells were incubated with the circINSR probes at 37°C overnight. Nuclei were stained with DAPI. Laser confocal microscopy was used to observe the localization of circINSR in cells (Nikon, Tokyo, Japan).

### RNA-Binding Protein Immunoprecipitation (RIP)

RNA-binding protein immunoprecipitation (RIP) assay was performed using the EZ-Magna RIP kit (#17-701, Millipore, Billerica, USA), according to the manufacturer's instructions. The cells were pelleted and resuspended in an equal pellet volume of RIP Lysis Buffer (~100 mL). The cell lysates (100 mL) were incubated with 5 mg beads coated with control mouse IgG or an antibody against Argonaute2 (Ago2, Abcam, UK) with rotation at 4°C overnight. After treatment with proteinase K, the RNAs were extracted with phenol: chloroform: isoamyl alcohol (125:24:1; pH, 4.5; #PD0419, Huyu Biotechnology, China) according to the steps in the Magna RIP kit. RNA was reverse transcribed using the Prime-Script RT Master Mix (TaKaRa, Japan), and the abundance of circINSR and miR-15/16 was detected by real-time qPCR.

### CircRNA Pull-Down

Biotin-labeled circINSR probe and negative control probe (NC probe) (RiboBio, Guangzhou, China) were used for circRNAs pull-down. We purchased the Pierce™ magnetic RNA-protein pull-down kit (#20164, Thermo, USA) and performed the experiment according to the manufacturer's instructions. In brief, the biotin-labeled probe was bound to streptavidin magnetic beads for 30 min. Then, the probe-labeled magnetic beads were incubated with the cell lysates from myogenic cells and preadipocytes for 1 h. Then, the RNA in the immunoprecipitates were eluted, extracted, and purified. Finally, real-time qPCR was used to detect the expression levels of circINSR and miR-15/16 in the immunoprecipitates.

### 5-Ethynyl-2′-deoxyuridine (EdU) and Cell Counting Kit-8 (CCK-8) Assay

When the density of myoblasts and preadipocytes reached 40–50%, transfection was performed with overexpression plasmid, siRNA, or miRNA mimics. After 24 h of transfection, cell proliferation was tested using an EdU assay kit (RiboBio, Guangzhou, China). The nucleus was stained with Hoechst 33342 (RiboBio, Guangzhou, China), and a fluorescence microscope was used to obtain images immediately after staining (AMG EVOS, Seattle, WA, USA). Similarly, we also used the CCK-8 (Multisciences, Hangzhou, China) assay to detect the level of cell proliferation after transfection. The optical density of CCK-8 at 450 nm was measured using an automatic microplate reader (Synergy4, BioTek, Winooski, USA).

### Cell Cycle and Apoptosis Assay

We used flow cytometry and the Cell Cycle Testing Kit (Multisciences, Hangzhou, China) to analyze the cell cycle. Myoblasts and preadipocytes were transfected when the cell growth density reached 50%. After transfection for 24 h the cells were collected and fixed overnight in 75% ethanol at 4°C. Subsequently, the kit instructions were followed to perform staining. Flow cytometry analysis was performed on a BD Accuri C6 now cytometer (FACS Canto™ II, BD Biosciences, USA), and data were processed using FlowJo7.6 software. Cell apoptosis assays were performed with the Annexin V-PE/7-AAD Apoptosis Detection Kit (RiboBio, Guangzhou, China) according to the manufacturer's recommendations. Afterward, the apoptosis rate was analyzed using flow cytometry (FACS Canto™ II, BD BioSciences, USA). Each treatment group was performed using three independent replicates.

### Western Blot Analysis

Proteins from cultured myoblasts and preadipocytes were lysed with RIPA buffer (Solarbio, Beijing, China). Proteins were separated by 12% sodium dodecyl sulfate-polyacrylamide gel electrophoresis (SDS-PAGE) and transferred onto polyvinylidene difluoride (PVDF) membranes (Thermo Fisher Scientific). The membranes were incubated overnight with primary antibodies specific for anti-GAPDH (1:1,000, #ab9485, Abcam, Cambridge, UK), anti-CyclinD1 (CCND1, 1:1,000, #ab226977, Abcam, Cambridge, UK), anti-Bcl-2 (1:500, #bs-0032R, Bioss, Beijing, China), anti-caspase-9 (1:500, #bs-0049R, Bioss, Beijing, China), anti-Bax (1:500, #bs-0127M, Bioss, Beijing, China), anti-fatty acid-binding protein (FABP4, 1:500, #bsm-51247M, Bioss, Beijing, China), anti-proliferating cell nuclear antigen (PCNA, 1:500, #WL01804, Wanlei Bio, Shenyang, China), anti-cyclin-dependent kinase (CDK2, 1:500, #WL01543, Wanlei Bio, Shenyang, China), anti-PPARγ (1:500, #WL01800, Wanlei Bio, Shenyang, China) and anti-C/EBPα (1:500, #WL01899, Wanlei Bio, Shenyang, China) at 4°C. The goat anti-mouse IgG (H&L)-horseradish peroxidase (HRP, 1:5,000, #bs-40296G, Bioss, China), and goat anti-rabbit IgG (H&L)-HRP (1:5,000, #bs-40295G, Bioss, China) were used as secondary antibodies. Protein bands were visualized using an enhanced chemiluminescence visualization system (ECL Plus, Amersham Life Sciences). The membranes were quantified with the chemiluminescence system (Bio-Rad, Hercules, CA, USA).

### Statistical Analyses

Data are expressed as the mean ± standard error (SEM) of at least three independent experiments. Statistical analyses were performed using SPSS 19.0 statistical software (SPSS, Chicago, IL, USA). Significant differences were determined using Student's *t*-test. A probability of 0.05 or less was considered statistically significant.

## Results

### Myoblasts and Intramuscular Preadipocytes Were Isolation From the Bovine Fetus

**Figure 1:**
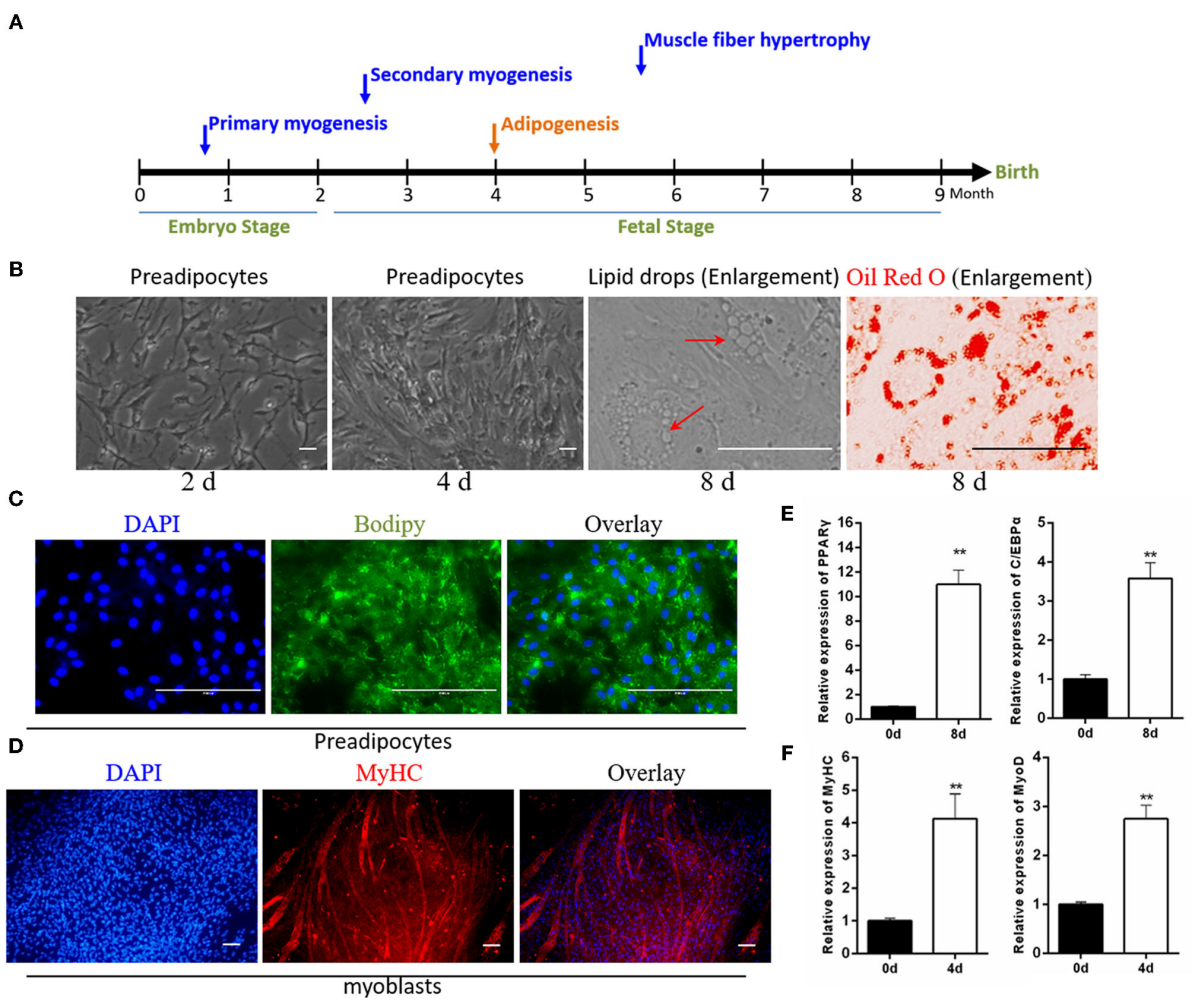
Identification of isolated myoblasts and intramuscular preadipocytes. **(A)** The growth pattern of muscle and fat during the embryonic development of cattle. **(B)** The growth morphology of isolated preadipocytes on days 2 and 4, and the Oil Red O staining of intracellular lipid droplets after 8 days of adipogenic differentiation. **(C)** BODIPY staining of preadipocytes after 8 days of adipogenic differentiation. **(D)** Anti-MyHC immunofluorescence staining of myoblasts after 4 days of myogenic differentiation. **(E)** The expression of adipogenic marker genes *PPAR*γ and *C/EBP*α after the differentiation of preadipocytes. **(F)** The expression of muscle differentiation marker genes *MyHC* and *MyoD* after the differentiation of myoblasts. Data are presented as the mean ± SEM. *n* = 3. **P < 0.01.

Skeletal muscle and intramuscular adipose tissue differentiate from mesodermal mesenchymal stem cells (MSCs) (Westerweel and Verhaar, 2008; Du et al., 2010a). The muscle begins to develop during the early embryonic period, and adipose tissue begins to develop during the second trimester (Du et al., 2010b) (Figure 1A). Enzymatic digestion, combined with the differential adhesion method, was able to roughly separate myoblasts from intramuscular preadipocytes. In this study, these isolated preadipocytes were spindle-shaped and possessed characteristics common to fibroblasts. After 8 days of adipogenic induction, small lipid droplets accumulated in some cells. The Oil Red O staining results also intuitively indicated that the isolated cells had undergone adipogenic differentiation (Figure 1B). The results of BODIPY staining showed that 8 days of adipogenic induction resulted in lipid accumulation in intramuscular preadipocytes (Figure 1C). In addition, the real-time qPCR results indicated the significantly increased expression levels of adipogenesis marker genes *PPAR*γ and *C/EBP*α in these cells (Figure 1E). Similarly, MyHC immunofluorescence showed that the isolated myoblasts were able to be induced into myotubes. The proportion of myotubes was analyzed by ImageJ software, showing ~35% differentiation (Figure 1D). Myogenic differentiation marker genes were also significantly overexpressed in these differentiated myotubes (Figure 1F).

### CircINSR Serves as a Sponge for miR-15/16

**Figure 2:**
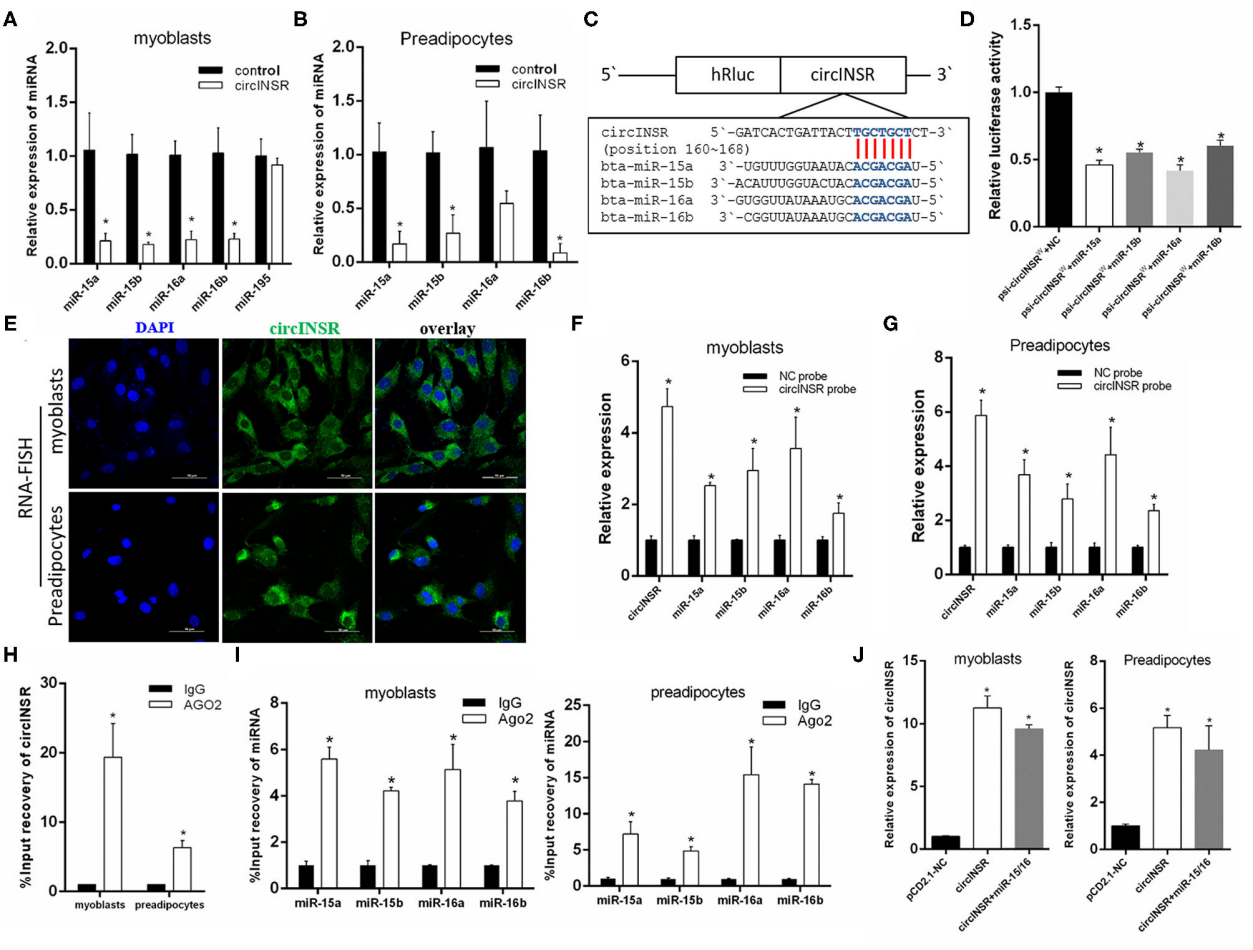
CircINSR sponges the miR-15/16 family in myoblasts and preadipocytes. **(A)** Changes in miR-15/16 family expression levels in myoblasts after the overexpression of circINSR. **(B)** Changes of miR-15/16 family expression levels in preadipocytes after the overexpression of circINSR. **(C,D)** Luciferase reporter activity of circINSR-WT in HEK-293T cells co-transfected with miR-15/16 mimics or mimic NC. **(E)** FISH detection of circINSR in myoblasts and preadipocytes. Scale bars, 50 μm. **(F,G)** CircRNA pull-down assays were performed using a specific biotin-labeled circINSR probe in myoblasts and preadipocytes. Real-time qPCR was used to detect the expression levels of circINSR and miR-15/16 family members in immunoprecipitates. *n* = 3. *P < 0.05 compared to negative control (NC) probe. **(H)** Ago2-RIP assay to assess the quantity of circINSR in myoblasts and preadipocytes. **(I)** Ago2-RIP assay to assess the quantity of miR-15/16 family members in myoblasts and preadipocytes. *n* = 3. *P < 0.05. **(J)** After overexpressing circINSR or co-transfecting circINSR with miR-15/16 in myoblasts and preadipocytes, real-time qPCR was used to detect the expression level of circINSR in cells. Data are presented as the mean ± SEM. *n* = 3. *P < 0.05.

Previous studies have shown that circINSR is able to adsorb miR-34a (Shen et al., 2020). According to the principle of adsorption between circRNAs and miRNAs, combined with prediction results based on high-throughput sequencing data, we found that circINSR also has the seed region binding site for the miR-15/16 family. In this study, after the overexpression of circINSR in myoblasts and preadipocytes, real-time qPCR results showed that the expression levels of miR-15a, miR-15b, miR-16a, and miR-16b were significantly reduced (Figures 2A,B). MiR-195 also belongs to the miR-15/16 family, but the expression level of miR-195 did not change following the overexpression of circINSR (Figure 2A). The binding sites for the miR-15/16 family on circINSR were predicted using Target Scan 7.0 and miRanda (Figure 2C). The results of the dual fluorescence reporter system showed that the overexpression of the miR-15/16 family significantly inhibited Renilla luciferase activity in the psi-CHECK2-circINSR^WT^ vector (Figure 2D). Because mature miRNA functions in the cytoplasm, circRNAs located in the cytoplasm are more likely to absorb miRNA (Hansen et al., 2013b; Barrett and Salzman, 2016). We used RNA-fluorescence *in situ* hybridization (FISH) probes targeting circINSR to analyze the subcellular localization of circINSR in myoblasts and preadipocytes. The results showed that circINSR was highly expressed in the cytoplasm of both cell types (Figure 2E). To determine the adsorption relationship between circINSR and miR-15/16, we designed a biotin-labeled circINSR probe. By taking advantage of the affinity between streptavidin magnetic beads and biotin probes, we conducted an RNA-pull down test. The results of the RNA-pull down assay revealed the high expression levels of circINSR and the presence of miR-15/16 family members in the circINSR immunoprecipitates from both myogenic cells and preadipocytes. A negative probe (NC probe) was used as a control (Figures 2F,G). To verify this adsorption, we performed an Ago2-RIP assay in both myoblasts and preadipocytes to detect whether both endogenous circINSR and miR-15/16 were bound to Ago2 protein. The results showed that the miR-15/16 family could be enriched by Ago2 protein pull-down from both myoblasts and preadipocytes (Figure 2I). The expression of circINSR was significantly higher in the Ago2 protein immunoprecipitation than in the IgG group (Figure 2H). To determine the accuracy of subsequent cell function tests, we overexpressed circINSR in myogenic cells and preadipocytes and examined the overexpression level of circINSR in cells co-transfected with circINSR and miR-15/16. The results show that the pCD2.1 vector can successfully overexpress circINSR in cells (Figure 2J).

### CircINSR Promotes Myoblast Proliferation and Inhibits Apoptosis

**Figure 3:**
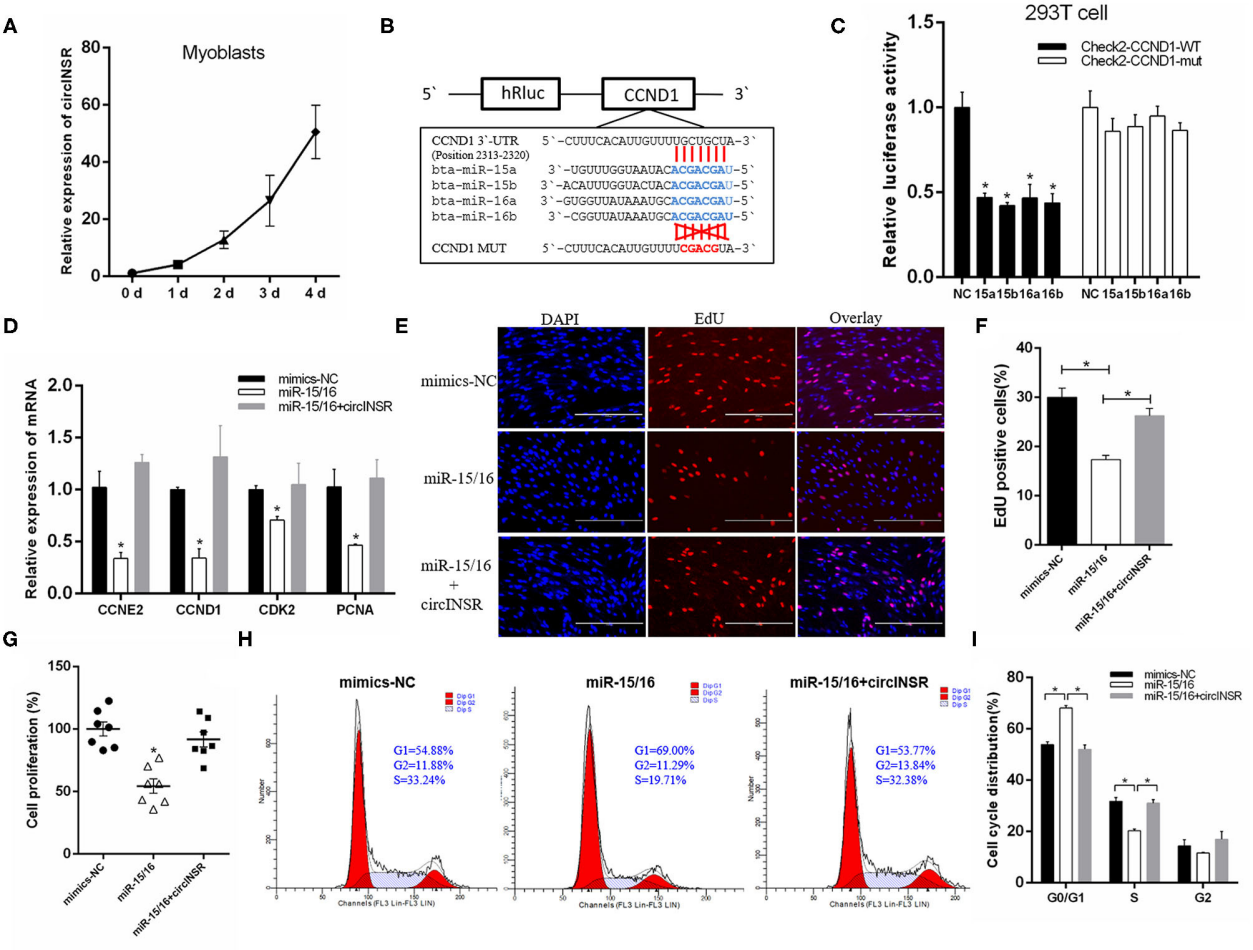
CircINSR promotes myoblasts proliferation by sponging the miR-15/16 family. **(A)** The expression of circINSR was detected by real-time qPCR during *in vitro* culture of myoblasts. **(B)** TargetScan predicted that *CCND1* 3'-UTRs contained binding sites for miR-15/16. **(C)** The fluorescence activity changes after co-transfection with dual fluorescent reporter vectors and miR-15/16. *n* = 3. **(D)** The expression of marker genes associated with cell proliferation after co-transfection with circINSR and miR-15/16 in myoblasts. *n* = 3. **(E,F)** EdU assay for myoblasts transfected with miR-15/16 mimics, either alone or with circINSR. Scale bars, 200 μm. *n* = 3. **(G)** CCK-8 assay for myoblasts transfected with miR-15/16 mimics, either alone or with circINSR. *n* = 6. **(H,I)** Cell cycle assay for myoblasts transfected with miR-15/16 mimics, either alone or with circINSR. *n* = 3. Data are presented as the mean ± SEM. *P < 0.05.

Our results showed that the expression level of circINSR in myoblasts gradually increases during *in vitro* culture, which implied that circINSR plays an important role in myoblast development (Figure 3A). Previous studies have reported that *CCND*1 and *Bcl-*2 are the target genes of the miR-15/16 family (Cimmino et al., 2005; Cai et al., 2012; Pekarsky et al., 2018; Cao et al., 2019). We inserted wild-type and mutant forms of the 3'-UTR sequence containing the miR-15/16 family binding site into the psi-CHECK2 vector to verify this binding using the dual fluorescent reporter gene system (Figure 3B). The results showed that the overexpression of the miR-15/16 family could significantly inhibit the activity of Renilla luciferase in the wild-type vector (Figure 3C). *CCND1* gene is a key regulator of cell proliferation. The real-time qPCR results showed that the overexpression of miR-15/16 mixed mimics could significantly reduce the expression levels of *CCND*1 and other cell proliferation marker genes. However, the co-transfection of circINSR and miR-15/16 was able to alleviate this inhibition (Figure 3D). The EdU assay results showed that the transfection of miR-15/16 mixed mimics was able to significantly reduce the number of proliferating cells, and co-transfection with circINSR was able to rescue this anti-proliferation effect (Figures 3E,F). CCK-8 cell proliferation analysis also obtained similar results (Figure 3G). The cell cycle results indicated that miR-15/16 blocked the cell cycle and reduced the number of cells that entered the S phase, whereas co-transfection with circINSR alleviated this inhibition (Figures 3H,I).

**Figure 4:**
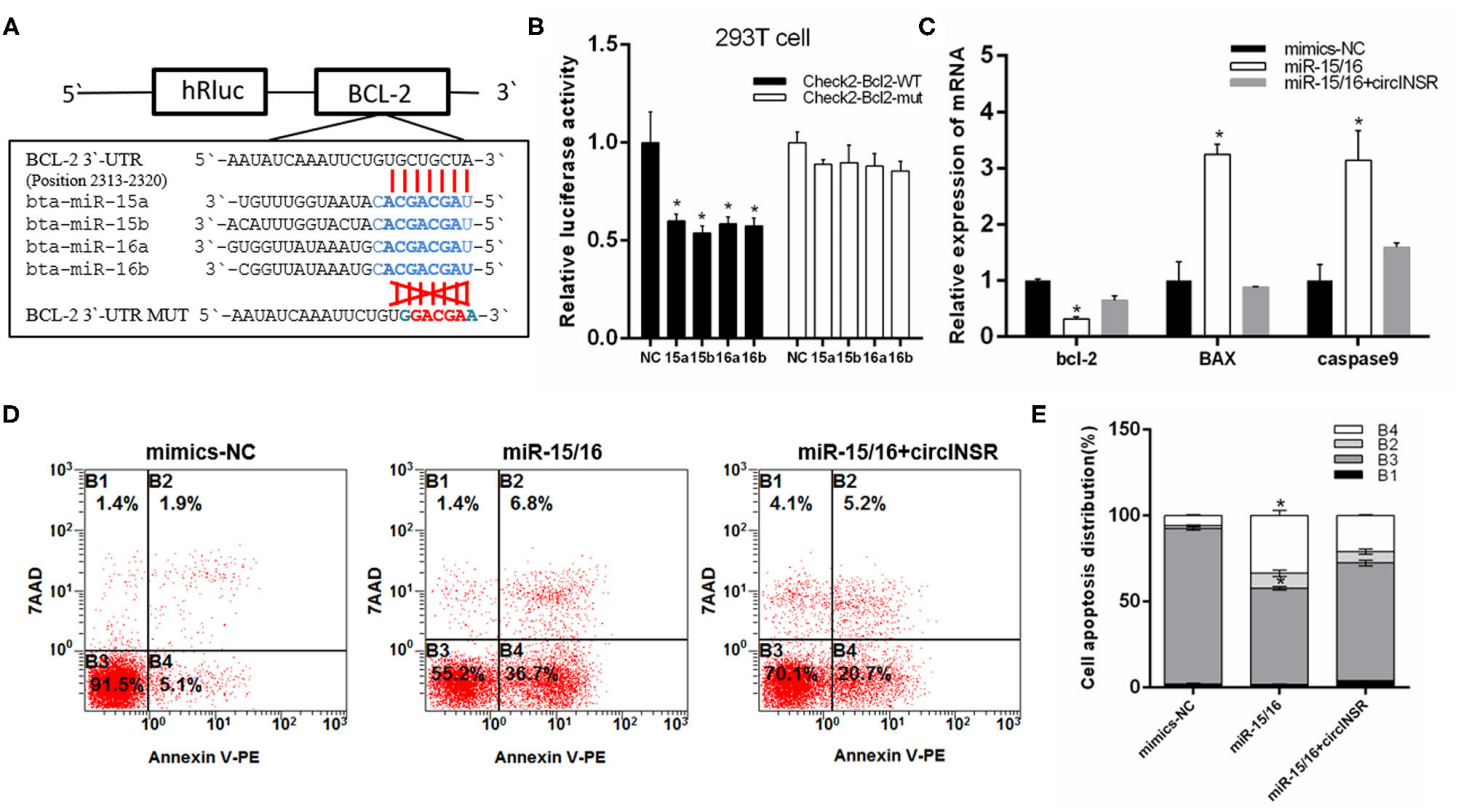
CircINSR inhibits the apoptosis of bovine primary myocytes by sponging the miR-15/16 family. **(A)** TargetScan predicted that *Bcl-2* 3'-UTRs contained binding sites for miR-15/16. **(B)** The fluorescence activity changes after co-transfection with dual fluorescent reporter vectors and miR-15/16. **(C)** The expression of marker genes associated with cell apoptosis after the co-transfection of circINSR and miR-15/16 in myoblasts. **(D,E)** Cell apoptosis was determined by Annexin V/7-AAD dual staining, followed by flow cytometry. B1: Annexin V^−^/7-AAD^+^, B2: Annexin V^+^/7-AAD^+^, B3: Annexin V^−^/7-AAD^−^, B4: Annexin V^+^/7-AAD^−^. *n* = 3. Data are presented as the mean ± SEM. *n* = 3. *P < 0.05.

To verify the targeting relationship between miR-15/16 and *Bcl-*2, we constructed wild-type and mutant psi-CHECK2 vectors (Figure 4A). The results of the fluorescence report analysis indicate that *Bcl-*2 was a potential target gene of miR-15/16 (Figure 4B). Real-time qPCR results showed that miR-15/16 significantly inhibited the expression of *Bcl-*2 and promoted the expression of apoptosis marker genes *Bax* and *Caspase*9 (Figure 4C). The subsequent flow cytometry analysis results showed that overexpression of miR-15/16 promoted myoblast apoptosis, whereas the co-transfection with circINSR reduced the number of apoptotic cells (Figures 4D,E).

### CircINSR Promotes Preadipocytes Proliferation by Sponging miR-15/16

**Figure 5:**
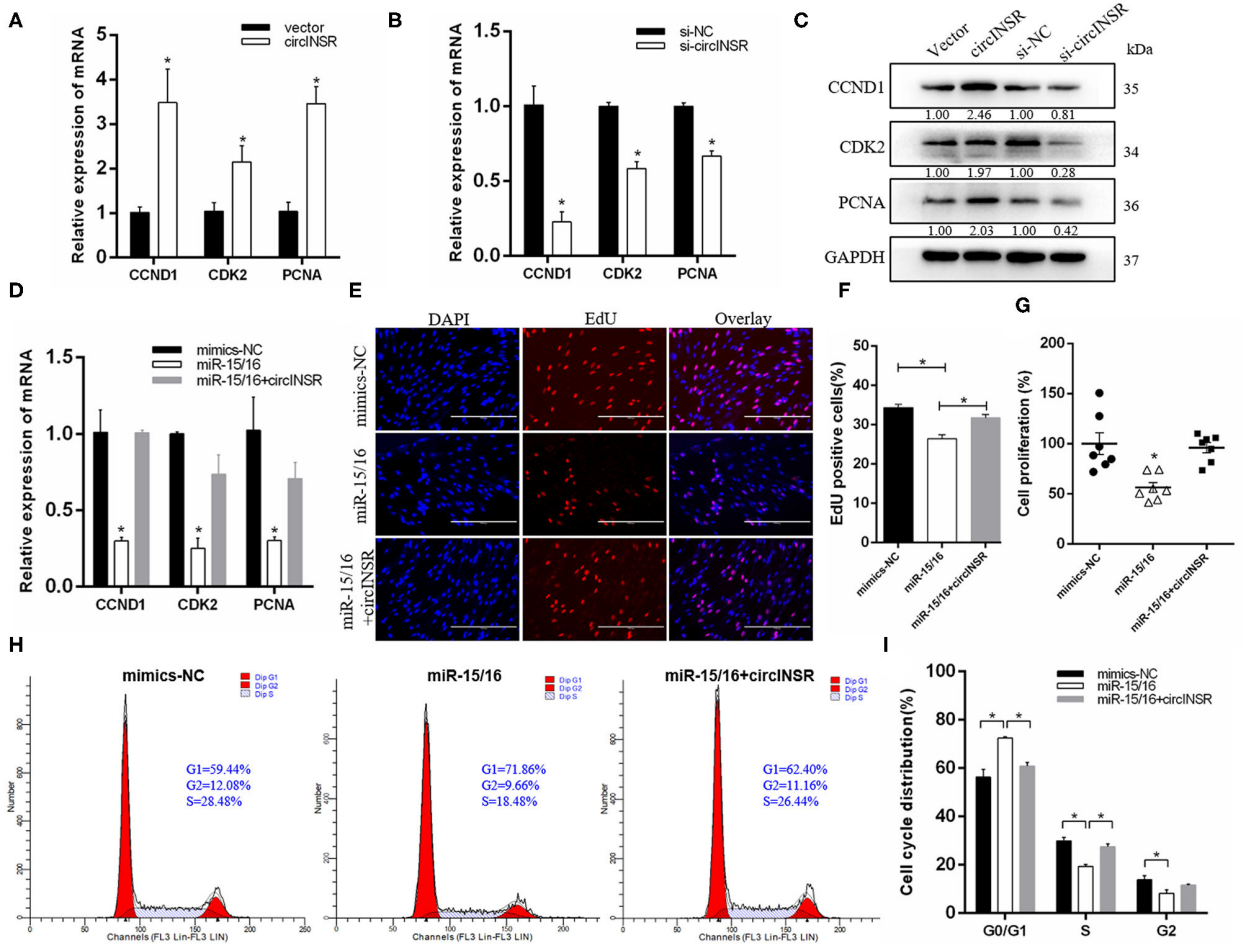
CircINSR promotes preadipocyte proliferation by sponging the miR-15/16 family. **(A,B)** The effects of circINSR overexpression and interference on proliferation marker genes in preadipocyte. *n* = 3. **(C)** The expression of CCND1, CDK2, and PCNA was detected by western blot analysis. **(D)** The expression of marker genes related to cell proliferation after co-transfection with circINSR and miR-15/16 in preadipocytes. *n* = 3. **(E,F)** EdU assay for preadipocytes transfected with miR-15/16 mimics, either alone or with circINSR. Scale bars, 200 μm. *n* = 3. **(G)** CCK-8 assay for preadipocytes transfected with miR-15/16 mimics, either alone or with circINSR. *n* = 6. **(H,I)** Cell cycle assay for preadipocytes transfected with miR-15/16 mimics, either alone or with circINSR. *n* = 3. Data are presented as the mean ± SEM. *P < 0.05.

To investigate the role played by circINSR during adipogenesis, preadipocytes were transfected with either the circINSR overexpression vector or si-circINSR. The results of real-time qPCR showed that the overexpression of circINSR significantly promoted the expression of cell proliferation marker genes (Figure 5A), whereas circINSR interference with circINSR inhibited the expression of these genes (Figure 5B). Western blot analysis revealed similar results (Figure 5C). In addition, the transfection of mixed miR-15/16 mimics in preadipocytes was able to significantly inhibit the expression of cell proliferation-related genes, whereas the co-transfection with circINSR was able to restore gene expression (Figure 5D). The results of the EdU (Figures 5E,F) and CCK-8 (Figure 5G) assays also showed that miR-15/16 inhibited cell proliferation, whereas the co-transfection of miR-15/16 and circINSR alleviated this inhibitory effect. Cell cycle assay showed that miR-15/16 inhibited preadipocytes from entering the S phase, whereas the co-transfection with circINSR promoted cell proliferation (Figures 5H,I).

### CircINSR Inhibits Preadipocyte Apoptosis by Sponging miR-15/16

**Figure 6:**
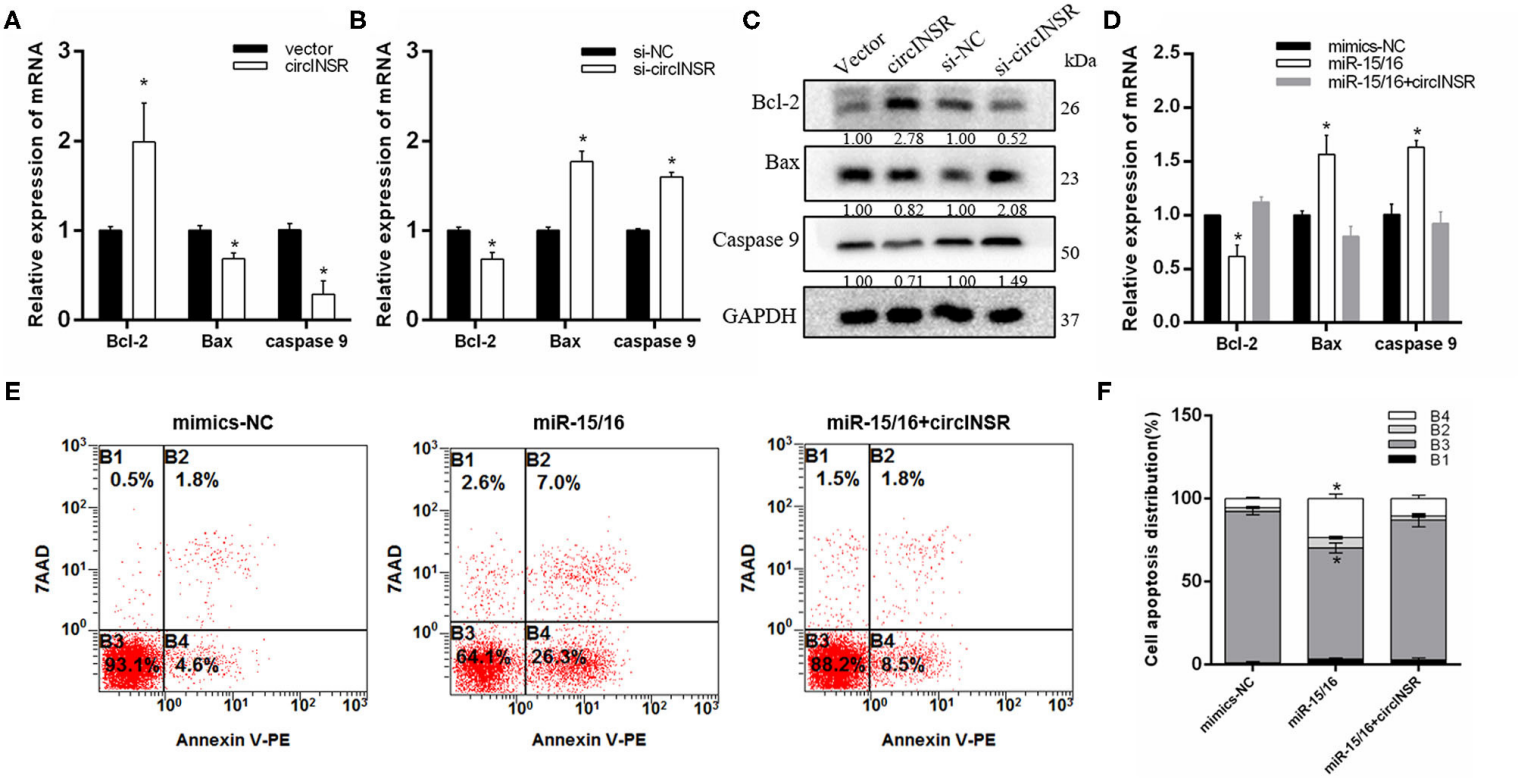
CircINSR inhibits preadipocytes apoptosis by sponging the miR-15/16 family. **(A,B)** The mRNA levels of cell apoptosis markers, including Bcl-2, Bax, and Caspase9, were detected by real-time qPCR in preadipocytes transfected with circINSR or siRNA. **(C)** The protein expression levels of Bcl-2, Bax, and Caspase9 were detected by western blot analysis. **(D)** The expression of marker genes associated with cell apoptosis after the co-transfection of circINSR and miR-15/16 in preadipocytes. **(E,F)** Cell apoptosis was determined by Annexin V/7-AAD dual staining, followed by flow cytometry. B1: Annexin V^−^/7-AAD^+^, B2: Annexin V^+^/7-AAD^+^, B3: Annexin V^−^/7-AAD, B4: Annexin V^+^/7-AAD. Data are presented as the mean ± SEM. n = 3. *P < 0.05.

To further explore the function of circINSR in preadipocytes apoptosis, real-time qPCR was used to detect the expression of apoptosis-related genes after either the overexpression or interference of circINSR. The results showed that circINSR promoted the expression of the anti-apoptotic gene *Bcl-2* and inhibited the expression of the pro-apoptotic genes *BAX* and *Caspase*9 (Figures 6A,B). The western blots analysis revealed a similar trend (Figure 6C). In contrast, real-time qPCR results showed that the overexpression of miR-15/16 inhibited *Bcl-*2 gene expression and promoted *BAX* and *caspase*9 expression. The co-transfection of miR-15/16 with circINSR inhibited the apoptosis of preadipocytes (Figure 6D). To further verify that circINSR could inhibit cell apoptosis by adsorbing the miR-15/16 family, we used Annexin V-PE/7-AAD staining combined with flow cytometry to analyze the effects of the co-transfection of miR-15/16 and circINSR on cell apoptosis. The results showed that miR-15/16 promoted cell apoptosis, whereas the co-transfection with circINSR rescued this anti-apoptotic effect (Figures 6E,F).

### CircINSR Inhibits Preadipocytes Differentiation

**Figure 7:**
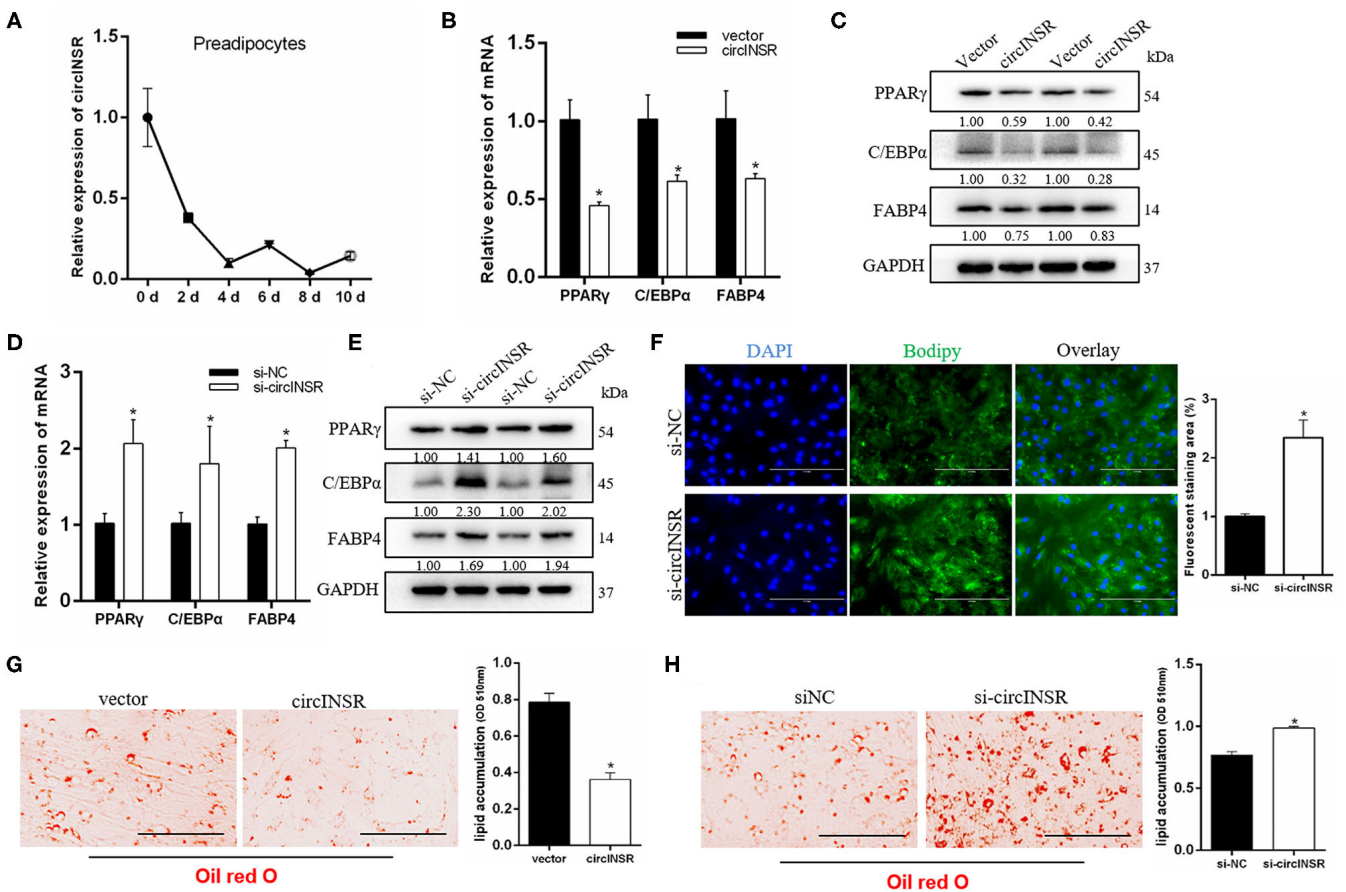
CircINSR inhibits adipogenic differentiation of preadipocytes. **(A)** The expression of circINSR was detected by real-time qPCR during preadipocytes differentiation. **(B,C)** The expression of adipogenic marker genes after the overexpression of circINSR in preadipocytes was detected by real-time qPCR and western blot analyses. **(D,E)** The expression of adipogenic marker genes after interference with circINSR in preadipocytes was detected by real-time qPCR and western blot analyses. **(F)** Interference with circINSR in preadipocytes, followed by BODIPY staining to analyze lipid droplet deposition. The fluorescence signal was analyzed by ImageJ software. **(G,H)** Lipid droplets in preadipocytes were stained with Oil Red O. Lipid contents were measured by spectrophotometric analysis after dissolution in isopropanol. Data are presented as the mean ± SEM. *n* = 3. *P < 0.05.

The above results suggested that circINSR could promote the proliferation of myoblasts and preadipocytes and inhibit cell apoptosis. Our results showed that the expression of circINSR gradually decreased during preadipocyte differentiation (Figure 7A). However, the expression of miR-15/16 family increased during the differentiation process (Supplementary Figure 1). We further studied the function of circINSR during preadipocyte differentiation. The results showed that the overexpression of circINSR was able to inhibit the expression of adipogenesis-related genes, including *PPAR*γ (Castillo et al., 1999), *C/EBP*α (Westerweel and Verhaar, 2008), and *FABP4* (Hoashi et al., 2008) (Figures 7B,C). In contrast, interference with circINSR promoted the expression of these genes (Figures 7D,E). BODIPY staining facilitates the direct observation of lipid droplet formation in adipocytes by monitoring green fluorescence. Because the circINSR overexpression vector carries a GFP cassette, we could only analyze BODIPY staining after circINSR interference and not under conditions of circINSR overexpression. Eight days after induction, the cells were subjected to BODIPY staining. The results showed that si-circINSR significantly increased the intensity of green fluorescence in preadipocytes (Figure 7F). In addition, Oil Red O staining results showed that overexpression of circINSR inhibited the lipogenesis of precursor fat, whereas the accumulation of lipid droplets increased after interference with circINSR (Figures 7G,H).

**Figure 8:**
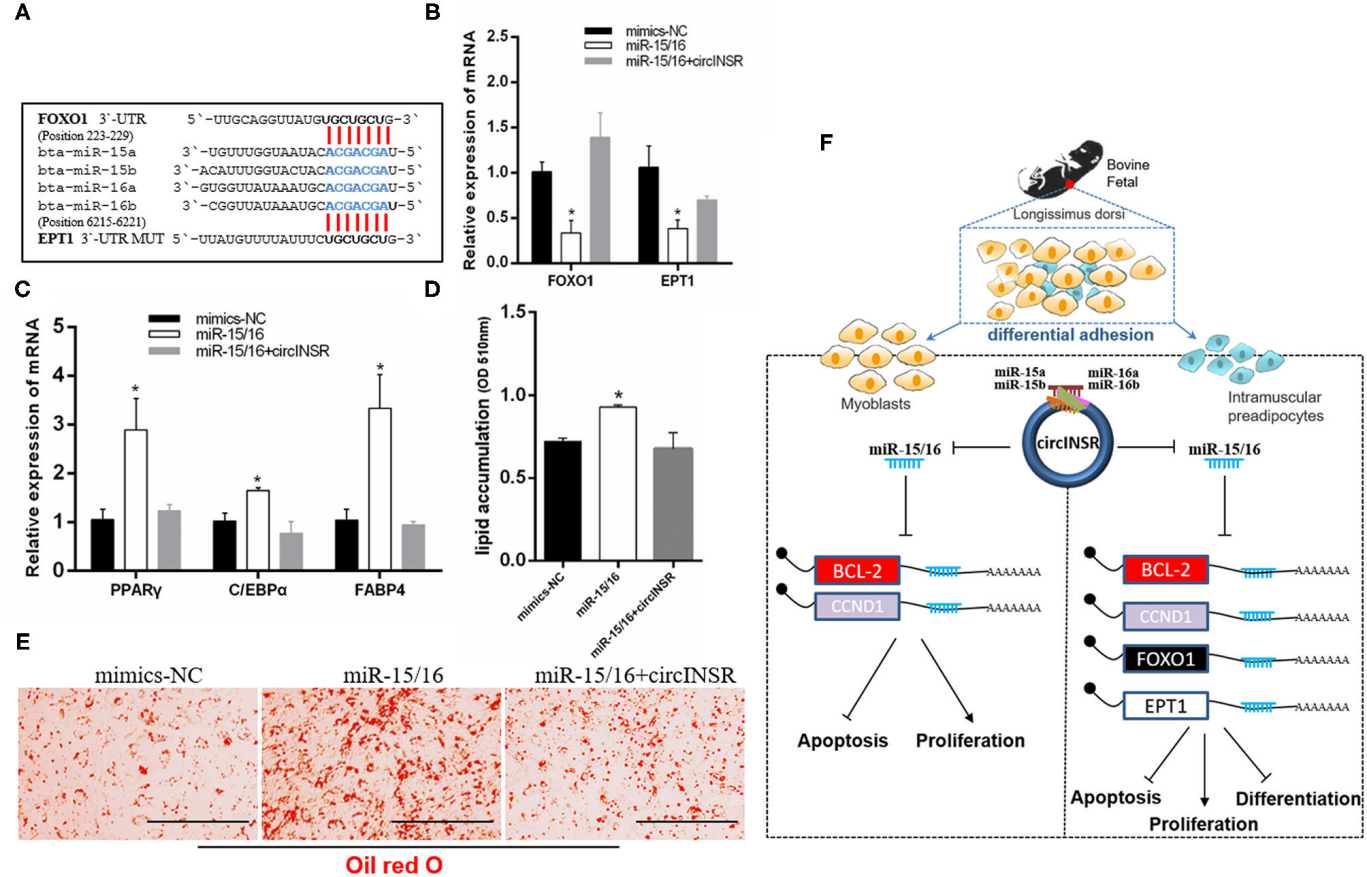
Adipogenic differentiation was regulated by circINSR through miR-15/16. **(A)** The reported target genes of the miR-15/16 family. **(B)** The effects of miR-15/16 transfection, either alone or with circINSR, on target genes in preadipocytes. **(C)** The expression of adipogenic marker genes in preadipocytes was detected by real-time qPCR. **(D,E)** Lipid droplets in preadipocytes were stained with Oil Red O. Lipid contents were measured by spectrophotometric analysis after dissolution in isopropanol. Data are presented as the mean ± SEM. *n* = 3. *P < 0.05. **(F)** Schematic diagram of the mechanisms through which circINSR regulates the proliferation, apoptosis, and differentiation in myoblasts and preadipocytes.

To further analyze whether the effect of circINSR on adipogenesis was related to miR-15/16, we identified two reported target genes for miR-15/16, Forkhead box protein O1 (*Foxo1)*, (Dong et al., 2014) and Ethanolamine phosphotransferase 1 (*EPT1*) (Xu et al., 2019) (Figure 8A). Real-time qPCR results showed that the expressions of *FOXO1* and *EPT1* were significantly reduced after miR-15/16 overexpression, whereas the co-transfection with circINSR rescued this inhibition (Figure 8B). In addition, miR-15/16 expression promoted the expression of adipogenic genes, whereas circINSR expression inhibited the expression of adipogenic genes (Figure 8C). The results of Oil Red O staining showed that miR-15/16 expression promoted lipid accumulation in preadipocytes, whereas the co-transfection with circINSR inhibited adipogenesis (Figures 8D,E).

## Discussion

Myoblasts and intramuscular adipocytes are derived from mesenchymal stem cells (MSCs) (Du et al., 2010a). As a result of complex signaling regulatory pathways, some MSCs will differentiate into myogenic and non-myogenic cell lines. Myogenic cells enter the process of muscle development, whereas non-myogenic cells enter the process of adipogenesis or fibroblast development (Du et al., 2010b; Stachecka et al., 2018). At ~180 days of gestation in bovine fetuses, IMF begins to appear, suggesting that adipogenic progenitor cells have already begun to differentiate into preadipocytes at this stage (Du et al., 2013). Therefore, we can separate myoblasts and intramuscular preadipocytes by using enzyme digestion combined with differential adhesion screening.

In this study, by strictly following the isolation and induced differentiation protocols that have been reported by previous authors (Beier et al., 2011; Miyake et al., 2012; Wang et al., 2015), we obtained myoblasts with high purity. The method of myoblast separation in this study has been widely used (Wei et al., 2017; Song et al., 2019; Wang et al., 2019). The paired box transcription factor *Pax7* is a key regulator of skeletal muscle stem cells and is required along with *Pax3* to generate skeletal muscle precursors (Seale et al., 2000; Relaix et al., 2005). Wang et al. reported that the primary myoblasts isolated using the same method could be stained with anti-PAX7 fluorescent antibody (Yi-Min et al., 2014). In this study, the thick myotubes could be labeled with anti-MyHC fluorescent antibody after 4 days of differentiation. However, the isolated preadipocytes contained MSCs, fibroblasts, and adipogenic progenitor cells, which affected cell purity. In studies of bovine intramuscular fat, adult beef is typically used as the starting material for preadipocyte isolation (Aso et al., 1995; Li et al., 2011). However, in our research, to study the parallel roles played by circINSR in the development of embryonic muscle and intramuscular fat, we chose to perform the cell isolation procedure during the embryonic stage. Existing research has indicated that the overall mass of adipose tissue increases with age in livestock (Hausman et al., 2009). Therefore, the number of embryonic preadipocytes was likely very small. Combining cell surface marker proteins (such as CD140a) and flow cytometry sorting might improve the purity of preadipocytes in future experiments (Guan et al., 2017). However, in view of the pluripotency of embryonic cell differentiation, under the adipogenic induction conditions in this study (100% cell density before differentiation), about 30% of the cells undergo adipogenic differentiation and form lipid droplets (Figure 8E). The preadipocytes were also successfully induced to differentiate, and lipid droplets could be identified using Oil Red O and BODIPY staining. Therefore, the cells obtained through our isolation and differentiation method could be used to explain the function of circINSR to a certain extent.

The development of muscle and fat is regulated by a complex signal network that involves both coding genes and non-coding RNAs. In this study, the expression levels of *PPAR*γ and *C/EBPa* in differentiated preadipocytes increased significantly. After 4 days of myogenic differentiation, *MyoD* and *MyHC* expression increased significantly. These coding genes play important roles in the differentiation of muscle and fat. In addition, an increasing number of reports have indicated that non-coding RNAs are also involved in the regulation of muscle and fat development (Sun et al., 2018; Li et al., 2019; Jiang et al., 2020).

CircINSR is highly homologous to the human has_circ_0048966, which is formed by the head-to-tail splicing of *INSR* exon 2 (552 bp), and is primarily expressed in the cytoplasm. In previous studies, circINSR has been shown to regulate cell proliferation and apoptosis through miR-34a-modulated *Bcl-*2 and *CyclinE*2 expression (Shen et al., 2020). In this study, we found that circINSR was able to sponge the miR-15/16 family. The real-time qPCR results showed that the overexpression of circINSR in myoblasts and preadipocytes could significantly inhibit the expression of the miR-15/16 family. After the co-transfection of the psi-CHECK2-circINSRWT vector and miR-15/16, the circINSR sequence, which contained in the 3'-UTR region of Renilla luciferase, was recognized by miR-15/16, affecting the translation of the Renilla gene and decreasing the Renilla: Firefly ratio in the final system, which indirectly supports a targeting relationship between circINSR and miR-15/16.

In addition, we used RNA pull-down and RNA-RIP technologies to verify the adsorption relationship between circINSR and the miR-15/16 family. First, we designed a biotin-labeled oligonucleotide probe for the specific linker sequence of circINSR. Using streptavidin-coated magnetic beads, we could precipitate and separate the protein and RNA complexes bound to circINSR. In this study, we detected high levels of miR-15/16 in the immunoprecipitation complex, further supporting that circINSR could adsorb the miR-15/16 family in cells. Ago2 (Argonaute 2) protein is the core component of the RNA-induced silencing complex (RISC), which acts on mature miRNA (Chendrimada et al., 2005). The Ago2 protein immunoprecipitation method is an important method used to isolate and identify miRNA target genes and is now widely used to verify the targeting relationship between circRNA and miRNA (Wang et al., 2018; Tang et al., 2019). In our study, the results of Ago2-RIP also illustrated the targeting relationship between circINSR and miR-15/16. Therefore, according to the molecular mechanism of sponging miRNAs, circRNAs should have the same targeting ability in view of the same seed sequence of miRNAs family.

In animals, single-stranded miRNAs bind to specific mRNAs through sequences that are imperfectly complementary to the target mRNAs, particularly those found in the 3'-UTR (Carthew and Sontheimer, 2009). Existing studies have reported the regulatory mechanism of *Bcl-*2 and *CCND*1 in cancer cells, including the post-transcriptional downregulation by miR-15 and miR-16 (Cimmino et al., 2005; Pekarsky and Croce, 2015; Pekarsky et al., 2018). In this study, we verified the interaction between miR-15/16 with *Bcl-*2 and *CCND*1. The overexpression of miR-15/16 in myoblasts and preadipocytes inhibited cell proliferation and promoted apoptosis. Additionally, the effects of miR-15/16 were counteracted when circINSR was co-transfected. These results indicated that during the embryonic stage, circINSR could promote muscle development and increase the number of intramuscular preadipocytes.

The number of intramuscular preadipocytes determines the degree of marbling that can occur during fattening. However, the pre-mature maturation of the IMF can result in fetal muscle insufficiency and the wasting of nutrition during pregnancy. For example, the early muscle tissue that develops during Duchenne muscular dystrophy is manifested as muscle fiber regeneration and mild lipid droplets; however, the late muscle fibers are gradually replaced by fat and connective tissue, with deleterious effects on muscle function (Foxley et al., 1991; Wren et al., 2008; Gaeta et al., 2012). Studies have indicated that miR-15/16 promotes adipogenesis by targeting *Foxo1* (Dong et al., 2014) and *EPT1* (Xu et al., 2019). Existing studies have revealed that *Foxo1* can inhibit the expression of *PPAR*γ to regulate the differentiation of adipocytes (Armoni et al., 2006; Wang and Tong, 2008). Dong et al. (2014) reported that miR-15a/b could promote adipogenesis by inhibiting the target gene *Foxo1*. *EPT*1, also known as selenoprotein 1 (*SELENO1*), encodes an enzyme that transfers phosphor-ethanolamine to produce ethanolamine glycerophospholipids (Horibata, 2018). Xu et al. (2019) reported that miR-16 regulates 3T3-L1 adipocyte differentiation through its target gene *EPT1*. In our results, the overexpression of circINSR in preadipocytes inhibited the expression of key genes associated with adipogenic differentiation and reduced lipid droplet formation. This circINSR-mediated inhibition of adipogenesis was achieved by sponging miR-15/16. Therefore, one function of circINSR may be to inhibit adipogenesis during the fetal period to maintain the normal muscle function while ensuring the development of a sufficient number of intramuscular preadipocytes.

In conclusion, our results revealed that circINSR could negatively regulate miR-15/16 family expression (Figure 8F). CircINSR promotes the proliferation of myoblasts and preadipocytes and inhibits apoptosis. CircINSR also serves to inhibit the differentiation of intramuscular preadipocytes from ensuring the normal development of embryonic muscle. These results provide potential molecular targets for improving beef production and molecular breeding.

## Data Availability Statement

The raw data supporting the conclusions of this article will be made available by the authors, without undue reservation.

## Ethics Statement

The animal study was reviewed and approved by the Animal Care Commission of the College of Veterinary Medicine, Northwest A&F University.

## Author Contributions

HC and XS designed research. XS, XZ, WR, and YH performed experiments and analyzed data. XS wrote the paper. CL and XL contributed new analytic tools. HC and JT helped modify the language of this manuscript. All authors contributed to the article and approved the submitted version.

## Conflict of Interest

The authors declare that the research was conducted in the absence of any commercial or financial relationships that could be construed as a potential conflict of interest.
