# Supplementary material for: CircINSR Regulates Fetal Bovine Muscle and Fat Development
Source: Front Cell Dev Biol. 2021 Jan 6;8:615638. doi: 10.3389/fcell.2020.615638 (PMC7815687; doi:10.3389/fcell.2020.615638)
Supplement: Supplementary file 1 [file Table_1.DOCX]

**Supplementary Information**

CircINSR regulates fetal bovine muscle and fat development

**Shen et al.**

**Supplementary Table 1.**

Specific primers used for real time qPCR

| Gene names | Primer |
| --- | --- |
| miR-15a RT | GTCGTATCCAGTGCAGGGTCCGAGGTATTCGCACTGGATACGACACAAAC |
| miR-15a F | GCGCTAGCAGCACATAATG |
| miR-15b RT | GTCGTATCCAGTGCAGGGTCCGAGGTATTCGCACTGGATACGACTGTAAA |
| miR-15b F | GGCTAGCAGCACATCATGG |
| miR-16a RT | GTCGTATCCAGTGCAGGGTCCGAGGTATTCGCACTGGATACGACCACCAA |
| miR-16a F | GCGCTAGCAGCACGTAAAT |
| miR-16b RT | GTCGTATCCAGTGCAGGGTCCGAGGTATTCGCACTGGATACGACGCCAAT |
| miR-16b F | GCGCTAGCAGCACGTAAAT |
| miR-15/16 R | CAGTGCAGGGTCCGAGGT |
| U6 RT/R1 | CGCTTCACGAATTTGCGTGTCAT |
| U6 F1 | GCTTCGGCAGCACATATACTAAAAT |
| circINSR qPCR F | CTGTCCGCATTGAGAAGAAC |
| circINSR qPCR R | CGGAAGTCCTCAGGTCTCGT |
| GAPDHF | CACTCACTCTTCTACCTT |
| GAPDHR | GCCAAATTCATTGTCGTA |
| CyclinE2 F | CGGGTCTGGCGAGGTTT |
| CyclinE2 R | CTTCTTGCGGGGAATCCGT |
| CyclinD1 F | CCGTCCATGCGGAAGATC |
| CyclinD1 R | CAGGAAGCGGTCCAGGTAG |
| CDK2 F | TCTTTGCTGAGATGGTGACCC |
| CDK2 R | CATCTTCATCCAGGGGAGGC |
| PCNA F | AACCTCACCAGCATGTCCAA |
| PCNA R | CCAACGTGTCCGCGTTATCT |
| Bcl-2 F | ATGACCGAGTACCTGAAC |
| Bcl-2 R | CATACAGCTCCACAAAGG |
| Bax F | GAGATGAATTGGACAGTAACA |
| Bax R | TTGAAGTTGCCGTCAGAA |
| Caspase 9 F | TGGTGGTCATCCTGTCTC |
| Caspase 9 R | CATCCATCTGTGCCATAAAC |
| PPARγ F | AGGATGGGGTCCTCATATCC |
| PPARγ R | GCGTTGAACTTCACAGCAAA |
| C/EBPα F | TGGACAAGAACAGCAACGAG |
| C/EBPα R | TTGTCACTGGTCAGCTCCAG |
| FABP4 F | ATTTCCTTCAAATTGGGCCAG |
| FABP4 R | TTCATGACACATTCCAGCACC |
| FOXO1 F | AGCGGGCTGGAAGAATTCAA |
| FOXO1 R | GTTGTTGTCCATGGATGCGG |
| EPT1 (SELENOI) F | ACAAGCATGTACCCGACTGG |
| EPT1 (SELENOI) R | CCCACAACCGCAGTCACTAT |

**Supplementary Figure.**

**
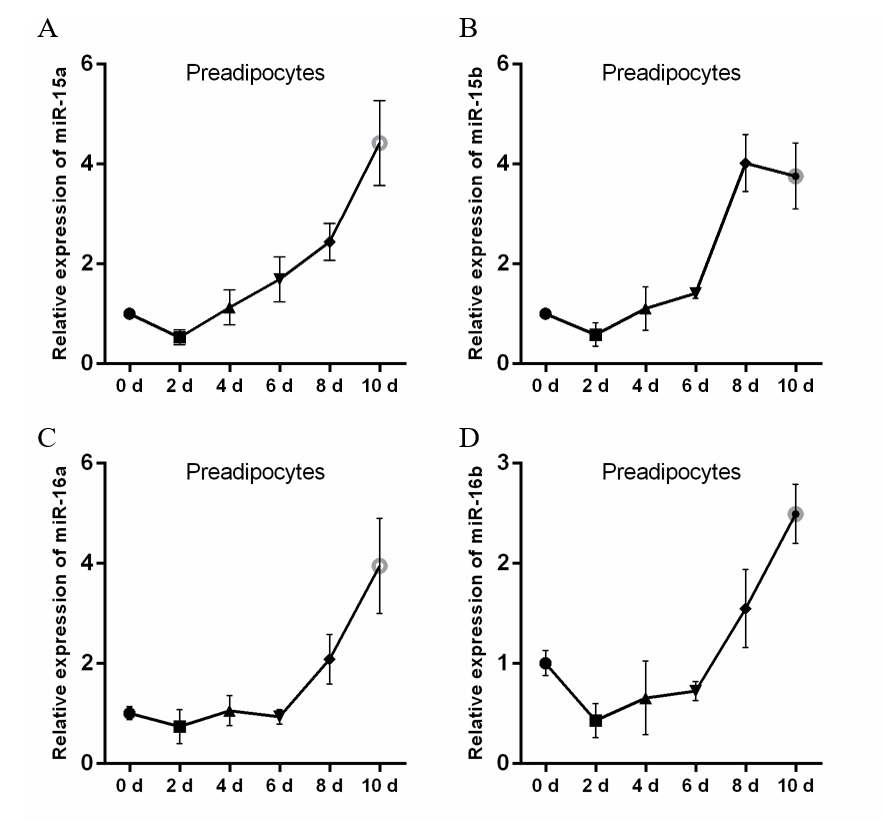
**

**Supplementary Figure 1.** (A-D) The expression of miR-15/16 family was detected by real-time qPCR during preadipocytes differentiation. Data are presented as the mean ± SEM. n = 3. **P* < 0.05.
